# Jejunal perforation secondary to migrated biliary stent

**DOI:** 10.1093/jscr/rjab057

**Published:** 2021-03-15

**Authors:** Renee Tabone, Peter Yuide, Matthew Burstow

**Affiliations:** General Surgery, Queensland Health, Logan Hospital, Meadowbrook, Queensland 4131, Australia; General Surgery, Metro South Health, Meadowbrook, Queensland 4131, Australia; General Surgery, Queensland Health, Logan Hospital, Meadowbrook, Queensland 4131, Australia; General Surgery, Queensland Health, Logan Hospital, Meadowbrook, Queensland 4131, Australia

## Abstract

An 80-year-old female presented with acute left-sided abdominal pain. Cross-sectional imaging demonstrated a contained perforation around a migrated biliary stent within a large incisional hernia. Significant surgical background included an open cholecystectomy complicated by bile leak and insertion of a biliary stent 2.5 years prior. The stent had migrated at the time of attempted retrieval 10 weeks post-insertion. A decision was made to pursue conservative management after which time she remained asymptomatic until her acute presentation. Emergency laparotomy, adhesiolysis, stent removal, small bowel resection and abdominal wall closure were successfully performed in this case. In the setting of the biliary stent migration, it is important to consider individual patient’s risk factors for acute perforation, such as intra-abdominal adhesions or diverticular disease, when deliberating conservative management versus elective surgical intervention for stent retrieval.

## INTRODUCTION

Cholecystectomy is one of the most common surgical procedures. Post-operative bile leak is a rare but serious complication of this procedure [1]. Historically, the management of post-cholecystectomy bile leak was with repeat surgical intervention; however, since the introduction of endoscopic retrograde cholangio-pancreatography (ERCP) [2], endoscopic stent placement is preferred, particularly in cases of bile leakage from the cystic duct or subvesical ducts. In this setting, the stent provides a conduit past the site of bile leakage, preventing extravasation of bile and allowing the defect to heal. It is retrieved endoscopically 6–8 weeks post-insertion [3]. In some cases, biliary stents migrate from their initial position. Migration can occur proximally into the hepatic ducts or distally into the gastrointestinal tract [4]. In rare instances, the migrated stent can result in gastrointestinal perforation. The duodenum is the most common site of such perforations; however, in rare cases, perforation at other gastrointestinal sites can occur particularly in patients with identified risk factors, such as adhesions or diverticular disease [5, 6]. This case report describes an acute clinical presentation of a migrated biliary stent causing jejunal perforation within a large incisional hernia and subsequent successful surgical intervention.

## CASE REPORT

This case pertains to an 80-year-old female who presented with acute left-sided abdominal pain. She had been treated in the community with oral amoxicillin and clavulanic acid for presumed diverticulitis. Her pain was associated with anorexia but was otherwise asymptomatic. Her surgical history included a Hartmann’s procedure for complicated diverticulitis, open Hartmann’s reversal and a previous open cholecystectomy 2.5 years prior for gangrenous cholecystitis with associated gram-negative sepsis. Her post-operative recovery from this procedure was complicated by a post-operative bile leak requiring ERCP, sphincterotomy and insertion of a 10 French plastic biliary stent. At the time of attempted stent removal 10 weeks post-operatively, the stent had migrated from the biliary tree. Small bowel enteroscopy was performed to the level of proximal jejunum; however, the stent was unable to be visualized. Subsequent imaging showed the stent positioned in the mid small bowel, with the relevant loop of small bowel located in a large incisional midline abdominal wall hernia. Her medical co-morbidities included previous B cell lymphoma in remission after completing chemotherapy in September 2019, moderate aortic stenosis, gastroesophageal reflux disease and osteoporosis. After extensive consultation in the outpatient setting regarding the risks and benefits of elective surgery, the patient opted to proceed with conservative management. This decision was based on the fact that she was asymptomatic, was still recovering from her lymphoma recurrence and did not want to undergo further major surgery in an elective setting. She remained asymptomatic for 2 years until the time of her emergency presentation.

**Figure 1:**
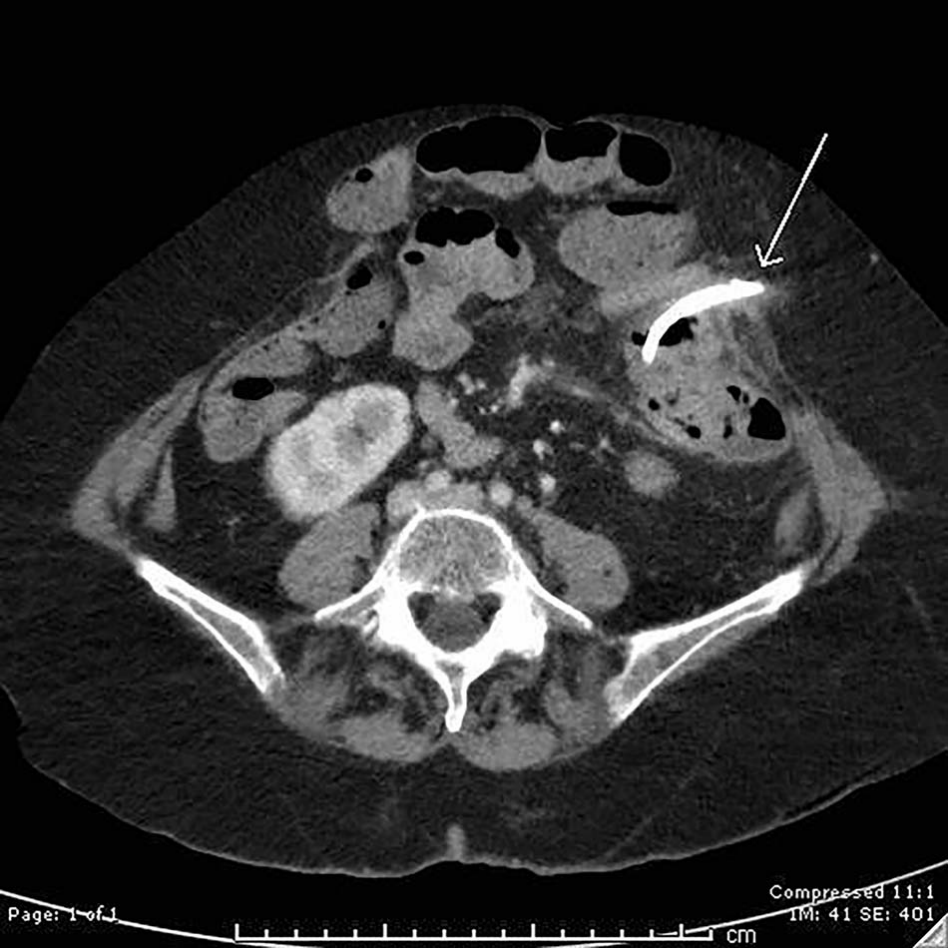
Axial computed tomography (CT) image demonstrating perforation of a migrated biliary stent into the anterior abdominal wall.

**Figure 2:**
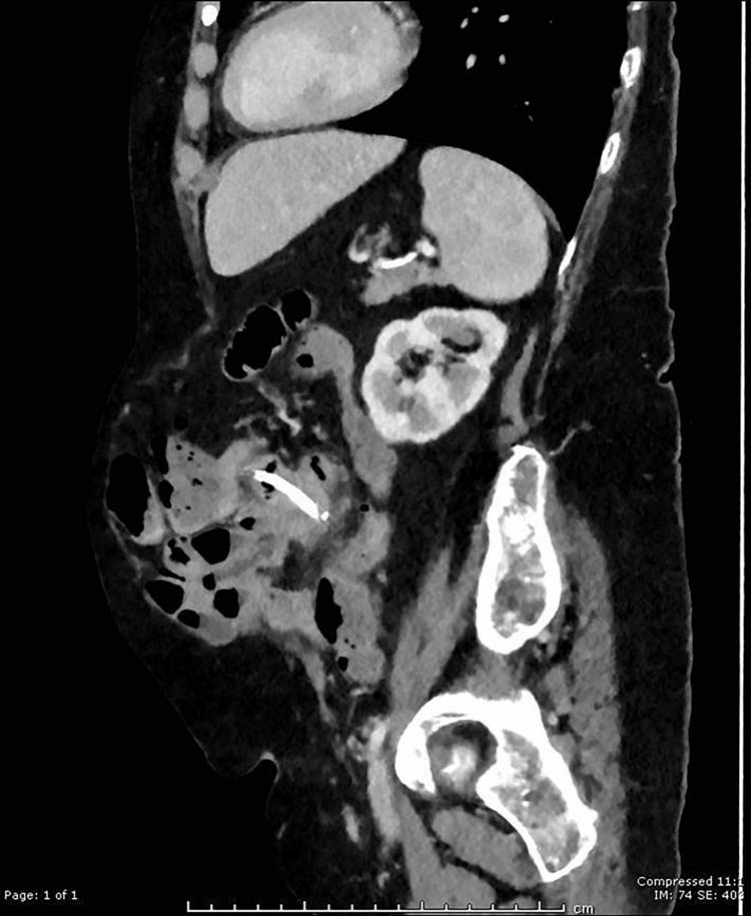
Sagittal CT image showing migrated biliary stent perforation through jejunum located within a large anterior abdominal wall hernia.

On examination, she had a large midline laparotomy scar, large reducible incisional hernia with localized peritonism in the left lower quadrant. She did not have generalized peritonitis. Biochemistry included a mild neutrophilia of 10.0 × 10^9^/l (reference range 2.0–8.0 × 10^9^/l), lactate of 2.6 mmol/l (reference range: 0.5–2.2 mmol/l) and C-reactive protein (CRP) of 125 mg/l (reference range < 2.0 mg/l). Cross-sectional imaging demonstrated a contained perforation around the migrated biliary stent within the incisional hernia (see Figs 1 and 2).

**Figure 3:**
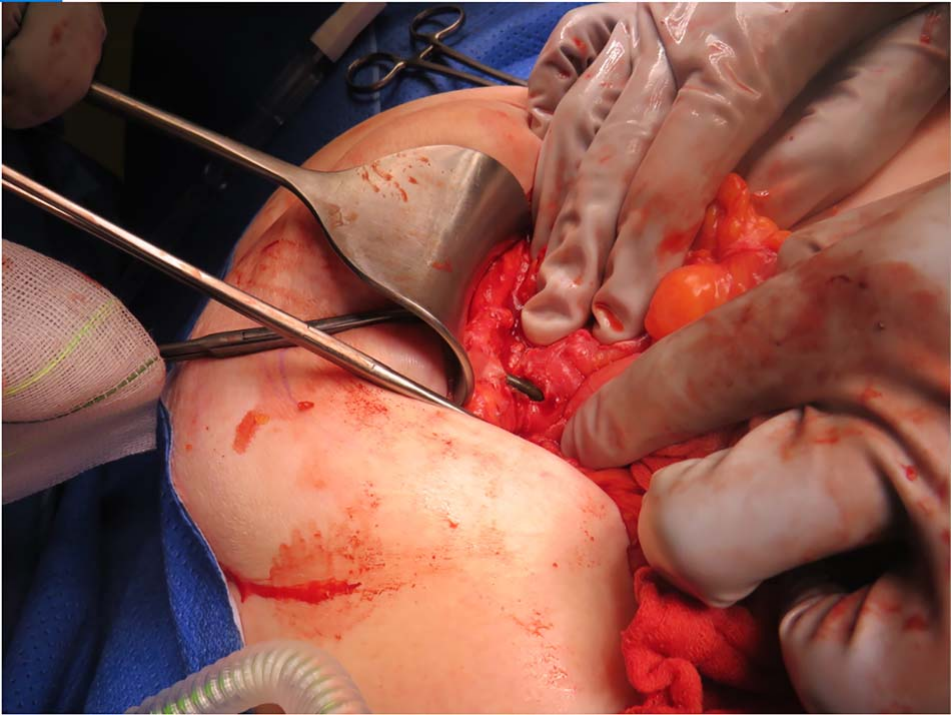
Intra-operative photograph demonstrating a biliary stent perforating through jejunum and lodging into the anterior abdominal wall.

The patient proceeded to emergency laparotomy, adhesiolysis, stent removal, small bowel resection and abdominal wall closure. Intra-operative findings confirmed that the biliary stent had eroded through small bowel within the large incisional hernia and into the anterior abdominal wall (see Fig. 3). Histopathology of the resected small bowel demonstrated mucosal ulceration, with a granulation tissue lined tract extending to the serosal surface consistent with perforation. The patient progressed well but slowly in the post-operative period with a prolonged post-operative ileus and issues with fluid balance. She required a 5-day intensive care admission in the context of respiratory distress secondary to fluid overload. During this time, she was also commenced on parenteral nutrition due to prolonged ileus and minimal oral intake. Her diet was gradually upgraded as her ileus resolved. Her parenteral nutrition was ceased on Day 40 of admission and she was discharged well on Day 42 post-operatively. She has been followed up in the outpatient setting and is currently recovering well.

## DISCUSSION

Bile leak is a well-recognized complication following cholecystectomy. ERCP and insertion of a temporary biliary stent is considered to be the optimal management for this complication and hence the use of ERCP and biliary stents is increasing [3]. Biliary stent migration occurs in 5–10% of patients [6]. Although stent migration is reasonably common, gastrointestinal perforation secondary to stent migration is rare with an incidence of less than 1% [7, 8]. The large majority of these perforations occur in the duodenum, with jejunal, ileal and colonic perforations being an even rarer entity.

Due to the low incidence of this serious complication, preventative strategies and optimal management strategies remain an area of debate. Risk factors for distal migration of biliary stents include the absence of malignancy, papillary stenosis, the use of straight stents (compared to pigtail), biliary sphincterotomy, common bile duct (CBD) diameter > 10mmm and the use of larger stents [4, 7, 9]. Risk factors for small intestinal perforation in the setting of a migrated biliary stent include any factors which create extrinsic fixation of the bowel, such as intra-abdominal adhesions. Risk factors for colonic perforation in the setting of stent migration include diverticular disease and strictures [5, 6]. Suggestion has been made that soft plastic pigtail stents opposed to straight plastic stents should be considered in patients with risk factors for perforation in setting of migration [5, 8–10]. Biliary stent removal is usually achievable via endoscopic methods. In cases where the stent has migrated beyond the ability to be retrieved endoscopically, the majority of stents will pass on their own and are hence managed conservatively [6].

This case highlights an uncommon scenario where a delayed intestinal perforation occurred beyond the duodenum in the setting of a migrated biliary stent. To our knowledge, very few other case reports have described this scenario, all in patients with identified risk factors such as intra-abdominal adhesions or abdominal wall hernias. It is important to keep this scenario in mind when considering whether or not to offer patients elective procedures to remove migrated biliary stents, particularly in the setting of risk factors for perforation.

## CONFLICT OF INTEREST STATEMENT

None declared.

## FUNDING

None.
